# Pigeon-Inspired Depth-Reasoning-Driven Decision Framework for Autonomous Traversal Flight of Quadrotors in Unmapped 3D Spaces

**DOI:** 10.3390/biomimetics11040283

**Published:** 2026-04-19

**Authors:** Yongbin Sun, Rongmao Su

**Affiliations:** 1 National Key Laboratory of Aircraft Integrated Flight Control, School of Automation Science and Electrical Engineering, Beihang University, Beijing 100083, China; rmsu@buaa.edu.cn; 2 State Key Laboratory of Autonomous Intelligent Unmanned Systems, Beijing 100083, China

**Keywords:** pigeon-inspired, Visual–Inertial Odometry, autonomous traversal flight, quadrotors, unmapped space, depth reasoning, constraint-fusion planning

## Abstract

Autonomous traversal flight in unknown 3D environments remains challenging due to mapping bottlenecks and computational latency. Inspired by pigeons navigating cluttered forests through instantaneous visual perception rather than constructing global metric maps, this paper presents a pigeon-inspired depth-reasoning-driven decision framework for agile quadrotor traversal in unmapped spaces without explicit map construction. To ensure feasibility, we leverage a robust state estimation backbone enhanced by deep-learning-based feature matching, providing stable pose feedback under aggressive maneuvers. The core contribution is a pigeon-inspired depth-reasoning framework that translates raw sensory depth data into a hybrid optimization framework, integrating both hard safety constraints and soft geometric smoothness constraints, directly emulating the three avian mechanisms: gap selection via instantaneous depth gradients, path selection that minimizes posture changes, and a safety field driven by the looming effect. By bypassing time-consuming mapping and spatial discretization processes, the framework significantly reduces perception-to-control latency. Finally, validated via simulations and real-world experiments on a resource-constrained quadrotor platform, our map-less approach achieves superior decision frequencies and comparable safety margins to those of state-of-the-art map-based planners. This framework offers a practical, high-frequency solution for autonomous flight where computational resources and environmental knowledge are strictly limited.

## 1. Introduction

With the ongoing advancement of aerial robotics, unmanned aerial vehicles (UAVs), especially quadrotors, have been increasingly involved in practical applications due to their remarkable maneuverability and flexibility. The potential of autonomous unmanned robots extends beyond quadcopters, permeating numerous fields. Significant theoretical progress has emerged, such as the development of VLM and VLA [1] in visual autonomous driving. Moreover, trajectory generation and quadcopter control [2] have become trending research topics. Nevertheless, unlike theoretical studies, the safety of unmanned systems in engineering applications has always been crucial for practical use. This highlights the importance of the generalization and robustness of system algorithms.

In practical applications, sensors are indispensable for continuously monitoring the external environment. Currently, a variety of sensors, including visual cameras, event cameras [3], LiDAR, and radar are widely utilized. Especially for exploration tasks in unmapped spaces, such as cave exploration, rubble search, and jungle traversal, flight planning under map-free conditions heavily relies on the external information captured by sensors, which directly impacts operational effectiveness. Each sensor type has distinct characteristics. While visual cameras offer more comprehensive information for subsequent tasks compared to other sensors, their positioning efficiency is inferior to that of radar sensors. However, the human navigation system heavily depends on the processing of visual information [4], such as visual cues and scene details, rather than pure positional data, to form a holistic spatial representation. This biological phenomenon finds a compelling parallel in nature: pigeons navigating through densely cluttered forests rely predominantly on instantaneous visual perception and optical flow analysis, constructing only an implicit spatial understanding rather than maintaining precise metric maps of their surroundings.

### 1.1. Related Works

#### 1.1.1. Visual–Inertial Odometry

In comparison to the Visual Odometry (VO) system that relies solely on visual positioning, the Visual–Inertial Odometry (VIO) system, which integrates an Inertial Measurement Unit (IMU), can achieve a pronounced complementary effect between the IMU and camera [5,6]. Equipped with the ability to capture high-frequency data, IMUs are particularly advantageous in short-term and fast-changing motions. Moreover, their low dependence on external environmental information endows them with high robustness across diverse environments. In contrast, visual positioning technology excels in long-term and slowly changing movements, enabling it to provide precise information when the environment remains relatively stable. The VINS-Mono algorithm [7,8] successfully integrated inertial navigation with visual positioning, effectively leveraging the strengths of both systems to enhance overall performance. Similarly, ORB-SLAM3 [9] began incorporating IMUs for fusion, thereby improving its own positioning capabilities. At the present stage, visual positioning technology has attained a certain level of maturity in terms of theory, with relevant theories being well-established. However, its application capability in real-world environments remains a significant challenge. The performance of visual technology is highly subject to environmental conditions, which is a major factor restricting its robustness.

Mainstream visual positioning techniques, including DSO [10], MSCKF [11], ROVIO [12], ICE-BA [13], and other algorithms, predominantly employ direct or optical flow methods to minimize the photometric error in the front-end odometer part to solve the least-squares optimization problem. While direct methods leverage per-pixel image information for camera pose estimation and reduce reliance on feature point extraction and matching, they partially circumvent issues from feature scarcity or matching inaccuracies. Nonetheless, the strong assumptions of photometric invariance and spatial continuity underpinning these methods may compromise convergence in real-world scenarios, particularly during visual positioning in crossing or highly maneuverable conditions.

ORB-SLAM [9,14,15] algorithms are based on indirect methods for front-end processing. Leveraging the descriptive and matching capabilities of ORB feature points, they effectively handle diverse complex real-world scenarios while meeting real-time requirements. Compared to other approaches, indirect methods rely on feature point matching [16,17,18,19,20] to acquire spatial information, offering a high degree of invariance. This enables them to provide superior feature information in more complex environments, making them more robust in practical applications. Recent advancements have increasingly leveraged deep learning to augment the robustness and information density of perception modules. DeepV2D [21] proposes a differentiable Structure-from-Motion module to estimate dense depth and camera motion from video streams. Meanwhile, DROID-SLAM [22] introduces a differentiable bundle adjustment layer to enable the recurrent iterative optimization of pose and depth estimates.

However, indirect methods also have drawbacks. The performance of indirect methods is closely tied to the quality of the feature points. In most cases, the visual positioning component provides status information for subsequent tasks. Since feature points represent sparse information, it is impossible to construct a dense map. This also complicates subsequent trajectory estimation tasks.

#### 1.1.2. Motion Planning

The soft-constrained trajectory planning method [2,23,24] incorporates flexible constraints into the trajectory planning process. This approach permits certain constraints to be violated within a defined scope to enhance other planning objectives, such as trajectory smoothness, energy efficiency, and time optimization. Nonetheless, it has notable drawbacks. It cannot strictly confirm that all constraints are satisfied and sensitive to parameter selection. This sensitivity means that environment-specific adjustments often require significant time and effort. Typically, this method assumes the environment is known and relatively static [25]. In complex or dynamic environments, soft constraints may not adapt quickly enough, leading to potential trajectory planning failures.

However, due to the low adjustability of hard constraints during the planning process, the generated paths cannot achieve better results than soft constraints, so the performance has always been mediocre. But this also means that in the actual execution process, the behavior of the trajectory planning system dominated by hard constraints is predictable, and there will be no violation of constraints. Moreover, due to the strictness of hard constraints, the planned trajectory is highly repeatable in multiple executions, which is very important for industrial production and automation tasks that require consistency [26]. Under the condition of satisfying hard constraints, optimization algorithms such as linear programming and nonlinear programming can be used to further optimize the performance indicators of the trajectory and improve the overall performance of the system. Currently, using reinforcement learning and other methods for trajectory planning [27,28] is also a popular direction, but the drawbacks are also very obvious. Under limited training conditions, it cannot adapt to complex environments and requires high investment in the early stages of the algorithm.

Prior map information or an established local cost map is necessary for trajectory planning [29]. For instance, CHOMP [30] leverages covariant gradient descent for continuous trajectory optimization, enabling the rapid generation of smooth, dynamically feasible trajectories. Similarly, optimization-based local planners such as FASTER [31] have significantly enhanced UAV safety and trajectory quality during high-speed flight by integrating safe backup trajectories with sampling strategies in free-known spaces. A majority of existing trajectory generation methods are fundamentally based on a soft constraint approach that is dominated by known environmental information from a third-party perspective. These methods focus on optimizing specific performance metrics using gradient-based optimization techniques. However, such approaches often result in trajectories with high redundancy and are unable to guarantee the safety of the planned trajectory in complex and unmapped spaces.

#### 1.1.3. Bio-Inspired Flight Behaviors

Beyond the engineering-oriented approaches discussed above, a rich body of ethological research has unveiled sophisticated flight strategies in birds that can directly inspire the design of autonomous UAVs [32]. Raptors, for instance, exhibit remarkable target tracking capabilities: they avoid the confusion effect in dense prey aggregations by locking onto fixed points, a strategy that ensures successful interception [33]. Similarly, hawks steer attacks using a guidance system specifically tuned for the close pursuit of erratically maneuvering targets [34].

Collective behavior in avian flocks also provides rich inspiration for multi-UAV coordination. Studies on mixed-species flocks of songbirds reveal that collective decision-making emerges from simple social interaction rules, enabling the group to respond adaptively to environmental changes [35]. Moreover, the presence of predators significantly influences flocking dynamics, leading to phase transitions in group behavior [36]. These findings underpin recent advances in bird-inspired self-propelled mechanisms for UAV swarm control [37], where phase transition principles are employed to achieve robust formation flight.

In addition to flocking and predation, individual flight maneuvers such as perching have been extensively studied. The optimization of avian perching maneuvers reveals fundamental principles of agility and precision, balancing kinetic energy management with trajectory planning [38]. Recent work on morphing-wing drones demonstrates how these biological strategies can be successfully replicated in engineered systems, achieving agile perching through wing articulation mechanisms inspired by birds [39].

### 1.2. Key Contributions of This Article

In the above context, autonomous exploration of unknown spaces by unmanned robots faces several challenges. First, current visual-information-based positioning methods are not robust and are greatly affected by the environment. This causes noticeable positioning errors and raises safety concerns in practical use. Second, with limited time and onboard computing resources, spatial mapping is delayed by another thread. This compromises the real-time nature of information. Existing work cannot ensure guidance using safe and reliable trajectories. Finally, current trajectory planning and optimization rely on known spatial information. The computational feasible domain in the environment is large, leading to high trajectory redundancy and heavy dependence on prior information. These challenges stand in stark contrast to the efficiency of biological flight—pigeons traverse unknown, cluttered environments at high speeds without explicit mapping, relying instead on a tight coupling between visual perception and motor control.

In this paper, inspired by this biological paradigm, the above problems are addressed through the design of a pigeon-inspired depth-reasoning-driven decision framework. Within the visual front-end, a deep learning framework is incorporated to enhance feature point matching. This is based on the indirect method, which is less susceptible to environmental influences, thereby bolstering the robustness of the visual positioning system.

In this work, we define unmapped navigation as a system that does not construct or maintain a globally consistent metric map (e.g., voxel grids, TSDF, or octomaps) for path planning. Instead, the system relies on a local instantaneous depth buffer and short-term visual–inertial states. This not only reduces computational load but also maintains positioning robustness, effectively addressing the non-real-time issue of dense mapping in delayed threads. For subsequent trajectory planning, a pigeon-inspired deep-vision-based autonomous navigation method is proposed. Drawing upon three mechanisms observed in pigeon flock flight—gap selection via instantaneous depth gradients [40], path selection that minimizes posture changes [41], and a safety field driven by the looming effect [42]—this method integrates depth perception with parametric trajectory generation, enabling quadrotors to navigate unmapped space and generate waypoints.

In our approach, hard constraints are primarily utilized while also integrating soft constraints. Based on the sparse path points obtained through the aforementioned method, we generate a controllable trajectory for the quadcopter’s safe flight. This enables autonomous and robust crossing flight of the quadcopter in a completely unknown complex environment. To validate its effectiveness, a series of experiments were designed and conducted. The contributions of this paper are as follows.

(1) A deep learning-based feature-matching state estimation method is integrated into the framework for autonomous traversal flight of quadrotors in unmapped 3D space, providing the necessary stability for the pigeon-inspired depth-reasoning-driven decision framework during maneuvering.

(2) A novel pigeon-inspired depth-reasoning-driven decision framework that directly generates trajectory constraints from raw sensory depth data. By bypassing explicit mapping and spatial discretization, which mirrors the avian ability to interpret visual scenes without constructing metric representations, the proposed method significantly reduces the latency from perception to control.

(3) A trajectory generation strategy integrating hybrid constraints, where raw depth information is transformed into hard safety boundaries, while soft smoothness terms are optimized to ensure the dynamic feasibility and continuity of the flight path in unmapped 3D spaces, which replicate the instantaneous trade-off between collision avoidance and flight efficiency observed in nature.

(4) Extensive comparative evaluations in high-fidelity simulations and feasibility validation on a resource-constrained physical platform.

The rest of the paper is organized as follows. Depth-guided VIO localization is introduced in Section 2. The pigeon-inspired depth-reasoning-driven decision framework is given in Section 3. The experimental results and analysis is discussed in Section 4. Section 5 summarizes this whole article.

## 2. Depth-Guided VIO Localization

The objective of VIO is to estimate the state of the mobile carrier by fusing data from the camera and IMU. Traditional VIO relies on geometric features, which perform poorly in weak texture environments, dynamic scenes, or intense motions, leading to reduced tracking reliability.

### 2.1. Problem Definition

**Figure 1:**
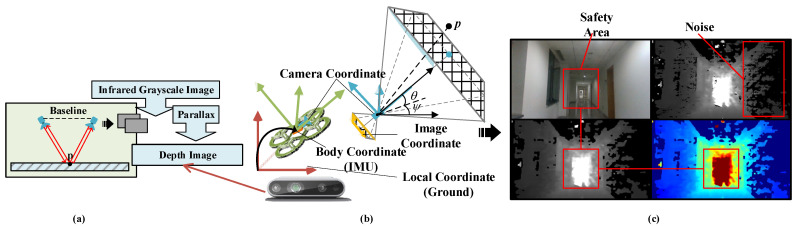
The state definition of the carrier and the sensor, the arrows in the figure represent the corresponding right-handed coordinate system. (**a**) Measurement principle diagram of the depth camera. (**b**) Some state information definitions in algorithms. (**c**) Image information received by the camera, including color images and depth maps.

As shown in Figure 1a, the infrared camera captures the infrared light reflected by an object to generate an infrared grayscale image. By comparing the images from the left and right infrared cameras, the disparity can be computed, which in turn allows for the determination of the distance between the object and the camera. Concurrently, the depth stream can be aligned with the color video stream to produce an image that contains both depth and color information.

We formalize the depth-guided VIO localization problem, which centers on fusing explicit depth measurements into the VIO framework to enhance robustness and accuracy. The objective is to devise a state estimation system that optimizes the integration of data from three complementary modalities: visual features Z, inertial measurements 𝓘, and depth information 𝒟. The system state defined at time t is

(1) $$x_{t}={\left[\begin{array}{llllll} p_{t} & v_{t} & q_{t} & b_{t}^{a} & b_{t}^{g} & f_{t}^{1:N} \end{array}\right]}^{T}$$

where $p_{t}\in {\mathbb{R}}^{3}$ is the position, $v_{t}\in {\mathbb{R}}^{3}$ is the velocity, $q_{t}\in SO\left(3\right)$ represents the attitude information, $b_{t}^{a}$ and $b_{t}^{g}$ are IMU accelerometer and gyroscope biases, and $f_{t}^{1:N}\in {\mathbb{R}}^{3}$ are the 3D coordinates of N tracked visual features. Given the following timing measurements.

(2) $$Z_{t}={\left\{z_{t}^{i}\right\}}_{i=1}^{N}={\left\{u_{t}^{i},v_{t}^{i}\right\}}_{i=1}^{N}$$

(3) $${𝓘}_{t}={\left\{a_{\tau },\omega_{\tau }\right\}}_{\tau =t_{0}}^{t}$$

(4) $${𝒟}_{t}={\left\{d_{t}^{j}\right\}}_{j=1}^{M}$$

where d is the depth value aligned with the visual feature, u and v are the pixel coordinates, a and ω are the acceleration and angular velocity values measured by the IMU. The state estimation problem can be reduced to the maximum a posteriori (MAP) estimation problem in a sliding window of states $\chi =\left\{x_{t-k},\ldots ,x_{t}\right\}$.

(5) $$\chi^{*}=\arg\underset{\chi }{\max}\left(\prod_{\tau =t-k}^{t}p\left(x_{\tau }|x_{\tau -1},{𝓘}_{\tau }\right)\prod_{i,j}p\left(z_{\tau }^{i}|x_{\tau },f^{i}\right)p\left(d_{\tau }^{j}|x_{\tau },f^{j}\right)\right)$$

where $p\left(x_{\tau }|x_{\tau -1},{𝓘}_{\tau }\right)$ represents the IMU motion prior, $p\left(z_{\tau }^{i}|x_{\tau },f^{i}\right)$ is the visual reprojection likelihood, and $p\left(d_{\tau }^{j}|x_{\tau },f^{j}\right)$ is the depth measurement likelihood.

### 2.2. Deep Learning Network-Based Vision

**Figure 2:**
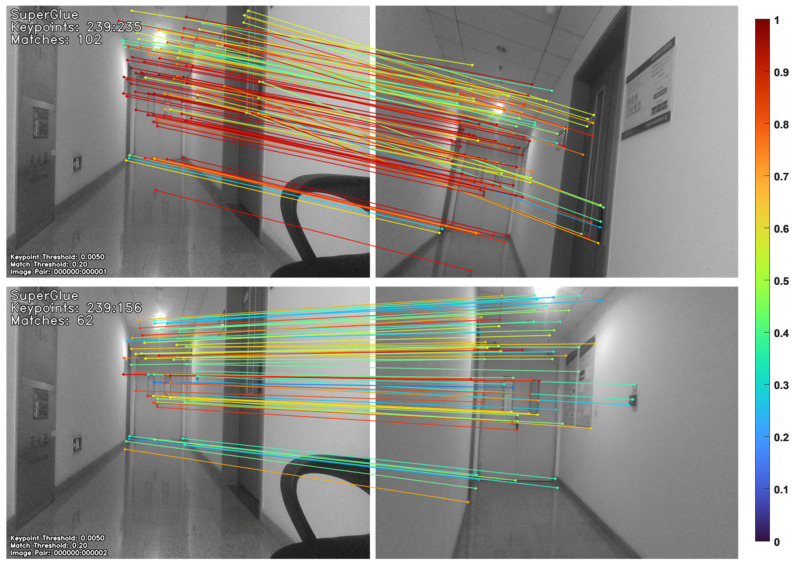
Feature extraction and matching results. The lines in the figure represent feature point pairs, and the colors represent confidence levels.

In our system, the visual–inertial state estimation is built upon a robust frontend utilizing SuperGlue [43,44] as shown in Figure 2. The decision to employ a learning-based matcher rather than traditional hand-crafted descriptors (e.g., ORB) is informed by its well-documented resilience to motion blur, extreme lighting variations, and low-texture environments—conditions that are frequently encountered during high-speed quadrotor flight. Extensive prior studies [45,46] have already demonstrated that deep learning network-based VIO significantly outperforms traditional methods in terms of tracking success rates and long-term stability. Consequently, we treat the VIO module as a robust perception backbone, focusing our investigation on how this stable state estimation enables high-frequency, map-less trajectory reasoning on resource-constrained hardware like the Jetson Xavier NX.

### 2.3. Sensor Fusion on SE(3) Manifold

#### 2.3.1. IMU Pre-Integration

The high-frequency characteristics of IMU data are not synchronous with those of low-frequency sensors like vision. Moreover, the measurements of IMU are susceptible to bias and noise. IMU Pre-integration [47] can pre-integrate IMU measurements between adjacent keyframes. It can generate observations decoupled from the initial state, thus greatly improving the efficiency of optimization. This section elaborates on the mathematical form of IMU pre-integration based on the theories of Lie groups and Lie algebras. Rotation group is $\mathrm{SO}\left(3\right)=\left\{R\in {\mathbb{R}}^{3}|R^{T}R=I,\det\left(R\right)=1\right\}$. Lie Algebra is so(3)={ϕ∈ℝ3|ϕ^∈ℝ3×3}, where ϕ^ is an antisymmetric matrix. Lie groups and Lie algebras satisfy the following exponential and logarithmic mappings.

(6) $$\begin{array}{cc} Exp\left(\phi \right)=R\in SO\left(3\right) & Log\left(R\right)=\phi \end{array}\in so\left(3\right)$$

The IMU measurement at time t is

(7) $$\left\{ \begin{array}{l} {\hat{\omega }}_{t}^{b}=\omega_{t}^{b}+b_{t}^{g}+n^{g}\\ {\hat{a}}_{t}^{b}=a_{t}^{b}+R_{w,t}^{b}g^{w}+b_{t}^{a}+n^{a} \end{array}\right.$$

where white noise $n_{a}∼N\left(0,\sigma_{a}^{2}\right)$ $n_{\omega }∼N\left(0,\sigma_{\omega }^{2}\right)$, g is the acceleration due to gravity. In the period $\left[t_{i},t_{j}\right]$, the incremental truth value discrete model of the state is

(8) $$\left\{ \begin{array}{c} R_{j}=R_{i}\cdot \prod_{k=i}^{j-1}Exp\left(\left({\hat{\omega }}_{k}-b_{k}^{g}-n_{k}^{g}\right)\Delta t\right)\\ v_{j}=v_{i}+g\Delta t_{ij}+\sum_{k=i}^{j-1}R_{k}\left({\hat{a}}_{k}-b_{k}^{a}-n_{k}^{a}\right)\Delta t\\ p_{j}=p_{i}+\sum_{k=i}^{j-1}\left[v_{k}\Delta t+\frac{1}{2}R_{k}\left({\hat{a}}_{k}-b_{k}^{a}-n_{k}^{a}\right)\Delta t^{2}\right] \end{array}\right.$$

where $\Delta t_{ij}=\sum_{k=i}^{j-1}\Delta t$. Introducing discrete IMU pre-integrated quantities decoupled from the initial state

(9) $$\left\{ \begin{array}{l} \Delta R_{ij}≜R_{i}^{T}R_{j}=\prod_{k=i}^{j-1}Exp\left(\left({\hat{\omega }}_{k}-b_{k}^{g}-n_{k}^{g}\right)\Delta t\right)\\ \Delta v_{ij}≜R_{i}^{T}\left(v_{j}-v_{i}-g\Delta t_{ij}\right)=\sum_{k=i}^{j-1}\Delta R_{ik}\left({\hat{a}}_{k}-b_{k}^{a}-n_{k}^{a}\right)\Delta t\\ \Delta p_{ij}≜R_{i}^{T}\left(p_{j}-p_{i}-v_{i}\Delta t_{ij}-\frac{1}{2}g\Delta t^{2}\right)=\sum_{k=i}^{j-1}\left[\Delta v_{ik}\Delta t+\frac{1}{2}\Delta R_{ik}\left({\hat{a}}_{k}-b_{k}^{a}-n_{k}^{a}\right)\Delta t^{2}\right] \end{array}\right.$$

The state deviation caused by the bias of the IMU is

(10) $$\left\{ \begin{array}{l} \Delta R_{ij}\left(b_{i}^{g}+\delta b_{i}^{g}\right)\approx \Delta R_{ij}\left(b_{i}^{g}\right)Exp\left(\frac{\partial R_{ij}}{\partial b_{i}^{g}}\delta b_{i}^{g}\right)\\ \Delta v_{ij}\left(b_{i}^{g}+\delta b_{i}^{g},b_{i}^{a}+\delta b_{i}^{a}\right)\approx \Delta v_{ij}\left(b_{i}^{g},b_{i}^{a}\right)+\frac{\partial \Delta v_{ij}}{\partial b_{i}^{g}}\delta b_{i}^{g}+\frac{\partial \Delta v_{ij}}{\partial b_{i}^{a}}\delta b_{i}^{a}\\ \Delta p_{ij}\left(b_{i}^{g}+\delta b_{i}^{g},b_{i}^{a}+\delta b_{i}^{a}\right)\approx \Delta p_{ij}\left(b_{i}^{g},b_{i}^{a}\right)+\frac{\partial \Delta p_{ij}}{\partial b_{i}^{g}}\delta b_{i}^{g}+\frac{\partial \Delta p_{ij}}{\partial b_{i}^{a}}\delta b_{i}^{a} \end{array}\right.$$

The recursive Jacobian matrix of the system state variable with bias is

(11) {∂Rij∂big=ΔRj,j−1∂Ri,j−1∂big−Jrj−1Δt∂vij∂big=∂vi,j−1∂big−[ΔRi,j−1(aj−1−bia)^∂ΔRi,j−1∂bigΔt]∂vij∂bia=∂vi,j−1∂bia−ΔRi,j−1Δt∂pij∂big=∂pi,j−1∂big+[∂vi,j−1∂bigΔt−12ΔRi,j−1(aj−1−bia)^∂ΔRi,j−1∂bigΔt2]∂pij∂bia=∂pi,j−1∂bia+[∂vi,j−1∂biaΔt−12ΔRi,j−1Δt2]

where $J_{r}\left(\cdot \right)$ is the Jacobian matrix of the right-multiplied small perturbation δϕ, using the BCH approximation.

(12) $$J_{r}^{k}=J_{r}\left(\left(\omega_{k}-b_{i}^{g}\right)\Delta t\right)$$

#### 2.3.2. Tightly Coupled Pose Estimation

**Figure 3:**
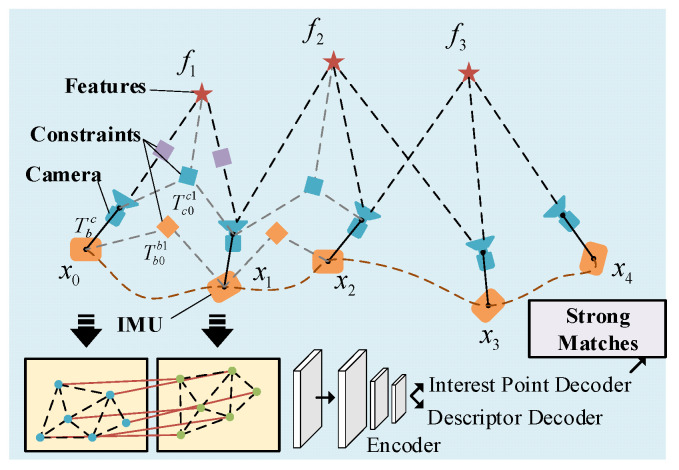
Information fusion based on tight coupling. The feature point information is fused into the estimation of IMU and depth information using a strong matching method.

As shown in Figure 3, a visual–inertial bundle adjustment (BA) framework is employed. To achieve the maximum a posteriori estimation, the sum of the prior and the Mahalanobis norm of all measurement residuals is minimized [48].

(13) $$\underset{\chi }{\min}\{{\left\|r_{p}-H_{p}\chi \right\|}^{2}+\sum_{k\in B}{\left\|r_{B}\left({\tilde{z}}_{b_{k+1}}^{b_{k}},\chi \right)\right\|}_{P_{b_{k+1}}^{b_{k}}}^{2}+\sum_{\left(i,j\right)\in C}\rho \left({\left\|r_{C}\left({\hat{z}}_{c_{i}}^{f_{j}},\chi \right)\right\|}_{P_{c_{i}}^{f_{j}}}^{2}\right)+\sum_{l\in D}\rho \left({\left\|r_{D}\left({\hat{z}}_{d_{l}}^{f_{l}},\chi \right)\right\|}^{2}\right)\}$$

where ρ(⋅) can be selected as either the Huber or Cauchy kernel function to target different levels of outliers. $r_{p}$ represents the residual of the prior information. $r_{B}$ represents the residual error estimated by the IMU. $r_{C}$ is the camera reprojection residual. $r_{D}$ is the residual of depth estimation. The specific definitions of each residual are as follows.

(14) $$r_{B}\left({\tilde{z}}_{b_{k+1}}^{b_{k}},\chi \right)=\left[\begin{array}{l} r_{\Delta R_{ij}}\\ r_{\Delta v_{ij}}\\ r_{\Delta p_{ij}}\\ \delta b^{a}\\ \delta b^{g} \end{array}\right]≜\left[\begin{array}{c} Log\left(\Delta {\hat{R}}_{ij}^{T}\Delta {\tilde{R}}_{ij}\right)\\ \Delta {\tilde{v}}_{ij}-\Delta {\hat{v}}_{ij}\\ \Delta {\tilde{p}}_{ij}-\Delta {\hat{p}}_{ij}\\ b_{k+1}^{a}-b_{k}^{a}\\ b_{k+1}^{g}-b_{k}^{g} \end{array}\right]$$

where ${\tilde{z}}_{b_{k+1}}^{b_{k}},\Delta {\tilde{R}}_{ij},\Delta {\tilde{v}}_{ij},\Delta {\tilde{p}}_{ij}$ ndicate the measurement value of IMU, $\Delta {\hat{R}}_{ij}^{T},\Delta {\hat{v}}_{ij},\Delta {\hat{p}}_{ij}$ represent the observation value of the camera, which serves as the reference value of the IMU data.

(15) $$r_{C}\left({\hat{z}}_{c_{i}}^{f_{j}},\chi \right)=\pi_{c}^{-1}\left(\left[\begin{array}{c} {\hat{u}}_{c_{i}}^{f_{j}}\\ {\hat{v}}_{c_{i}}^{f_{j}} \end{array}\right]\right)-T_{b}^{c}T_{w}^{b_{i^{\prime }}}T_{b_{i}}^{w}T_{c}^{b}\frac{1}{\lambda_{f_{j}}}\pi_{c}^{-1}\left(\left[\begin{array}{c} {\hat{u}}_{c_{i^{\prime }}}^{f_{j}}\\ {\hat{v}}_{c_{i^{\prime }}}^{f_{j}} \end{array}\right]\right)$$

where $\pi_{c}^{-1}$ is the back-projection matrix of camera. $\lambda_{f_{j}}$ is the estimated value of the depth of the feature point. $\left[\begin{array}{cc} {\hat{u}}_{c_{i}}^{f_{j}} & {\hat{v}}_{c_{i}}^{f_{j}} \end{array}\right]$ and $\left[\begin{array}{cc} {\hat{u}}_{c_{i^{\prime }}}^{f_{j}} & {\hat{v}}_{c_{i^{\prime }}}^{f_{j}} \end{array}\right]$ are the observed pixel coordinates of the consensus point for the i time and other frames, respectively.

(16) $$r_{D}\left({\hat{z}}_{d_{l}}^{f_{l}},\chi \right)=\lambda_{f_{l}}-d_{l}$$

where $d_{l}$ is the depth of the feature points measured by the depth camera.

## 3. Pigeon-Inspired Depth-Reasoning-Driven Decision Framework

**Figure 4:**
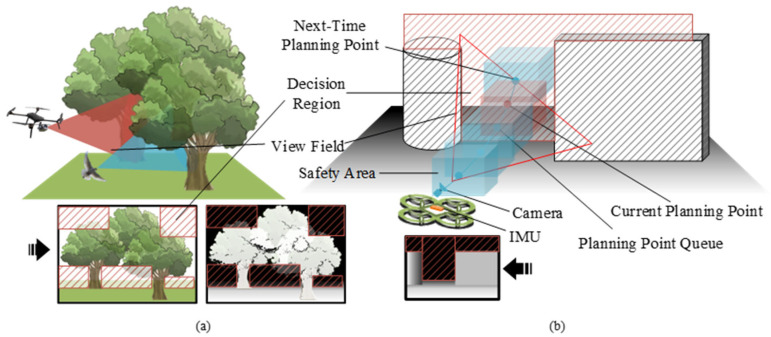
Pigeon-inspired vision-guided autonomous trajectory traversal of a quadrotor. (**a**) Similarity between quadcopter and pigeon vision in practice. (**b**) Reference state of the quadrotor during obstacle avoidance.

When executing flight maneuvers, avian flocks frequently encounter intricate environmental configurations, as depicted in Figure 4a. Under such circumstances, the necessity for rapid, robust, and safe flight is paramount. Consequently, birds rely solely on visual information to make local behavioral decisions. These mechanisms allow pigeons to perceive the looming effect of obstacles and identify traversable gaps through instantaneous depth-gradient reasoning. Similarly, in the context of quadrotor crossing flight, the visual guidance framework we have developed can rapidly delineate the decision-making area and generate a flight decision trajectory based on prevailing state information, as illustrated in Figure 4b. This capability, directly inspired by the pigeon’s real-time visual processing for gap detection, enables the execution of autonomous crossing flight missions within unmapped and complex spatial environments.

### 3.1. Basic Vision Model

Firstly, some information of the visual model and used quadrotor model are defined. During the entire flight process, the following four coordinate systems are used: the local coordinate system $p_{w}$ fixed to the ground, the body coordinate system $p_{b}$, the camera coordinate system $p_{c}$, and the imaging coordinate system $p_{uv}$, as shown in Figure 1b. The correlation between the coordinate systems is shown in the following formula. T and $K_{c}$ are the external parameter matrix and internal parameter matrix of the camera, respectively.

(17) $$\begin{array}{cc} p_{c}=T_{w}^{c}\cdot p_{w}, & T=\left[\begin{array}{cc} R_{w}^{c} & t_{w}^{c}\\ 0^{T} & 1 \end{array}\right] \end{array}$$

(18) $$\begin{array}{cc} p_{uv}=K_{c}\cdot p_{c}, & K_{c}=\left[\begin{array}{ccc} f_{x} & 0 & c_{x}\\ 0 & f_{y} & c_{y}\\ 0 & 0 & 1 \end{array}\right] \end{array}$$

The camera parameter vector are as Equation (19), where the horizontal and vertical focal length are $f_{x},f_{y}$, offset denote $c_{x},c_{y}$, field of view are $FOV_{x},FOV_{y}$, and depth detection range present $d_{\min},d_{\max}$.

(19) $$C=\left\{\begin{array}{ccc} f_{x} & \begin{array}{ccc} f_{y} & c_{x} & c_{y} \end{array} & \begin{array}{cc} FOV_{x} & \begin{array}{ccc} FOV_{y} & d_{\min} & d_{\max} \end{array} \end{array} \end{array}\right\}$$

During the flight of a quadrotor, Euler angles Θ, encompassing the roll angle φ, pitch angle θ, and yaw angle ψ, serve often as the conventional parameters for denoting the attitude information. During the acquisition of visual information by the front-mounted camera, the roll angle merely induces an in-plane rotation of the captured image without altering its spatial information. Once the image undergoes horizontal correction based on the roll angle, the pitch angle and yaw angle alone are considered in the ensuing visual guidance computation.

(20) $$\{\begin{array}{l} \Theta =\left\{\begin{array}{ccc} \varphi & \theta & \psi \end{array}\right\}\\ \Theta^{\prime }=\left\{\begin{array}{cc} \theta & \psi \end{array}\right\} \end{array}$$

(21) $$\chi_{t}=\left\{\begin{array}{cc} p & \Theta^{\prime } \end{array}\right\}=\left\{\begin{array}{cccc} x & y & z & \begin{array}{cc} \theta & \psi \end{array} \end{array}\right\}$$

In accordance with the camera’s parameters, specifically the field of view angle $FOV_{c}$ and depth detection range $d_{c}$, the field-of-view cone can be defined as follows.

(22) $$\left\{ \begin{array}{c} FOV_{c}=\left[-FOV_{x}/2,+FOV_{x}/2\right]\times \left[-FOV_{y}/2,+FOV_{y}/2\right]\\ d_{c}=\left[d_{\min},d_{\max}\right]\\ p_{c}\in \Omega_{c}=d_{c}\cdot e^{i\cdot FOV_{c}} \end{array}\right.$$

Obstacles within the field-of-view cone are observable and can participate in the current decision-making process. At the current moment, the quadrotor processes external visual information $I_{t}$, integrates it with its own state $\chi_{t}$ and the expected point position $\tilde{p}$, and outputs the expected decision state ${\tilde{\chi }}_{t}$. Figure 1c presents the data captured by a real-world depth camera. In practical applications, the captured image undergoes filtering and processing to enhance its usability.

(23) $$\begin{array}{cc} {\tilde{\chi }}_{t}=f\left(\chi_{t},I_{t},\tilde{p}\right) & I_{t}:\Omega_{img}\to \mathbb{R} \end{array}$$

**Figure 5:**
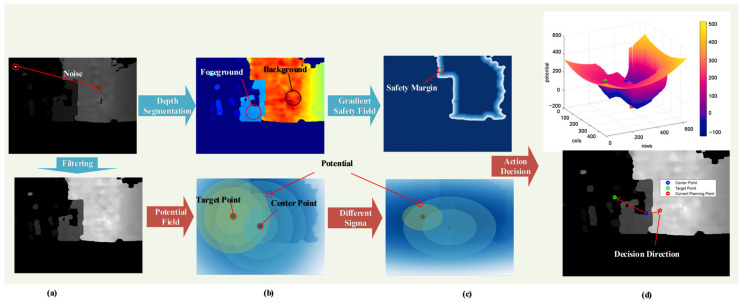
The processing and decision-making process of visual information in pigeon-inspired depth-reasoning-driven decision framework. (**a**) Depth image input by the original camera: The input information that can be used after depth information filtering. (**b**) Foreground and background of threshold segmentation after pseudo color display: The input information that can be used after depth information filtering. (**c**) Image of safety gradient field function and the image of non-uniform equipotential field function. (**d**) Decision potential field functions and results.

At its core, the obstacle avoidance strategy we proposed hinges on visual data to reroute around obstacles on the direct path from the current position to the target position. Depth information can easily separate the information in the image into foreground and background, as shown in Figure 5b. As shown in Figure 4b and Figure 5b, visual data enables the identification of obstacle-free regions, the creation of safe flight areas for quadcopters, and the division of the decision-making space.

### 3.2. Behavioral Constraints Judgment

Pigeons instantaneously identify passable gaps by analyzing depth gradients on the retina. Like the pigeon’s binocular disparity processing, we perform a real-time thresholding of the depth map to separate obstacles (foreground) from free space (background). The resulting binary mask is then analyzed to identify passable gaps without constructing any global map. The image will provide information about the location of obstacles. By integrating the quadrotor’s current position with the target point’s position, many logical decisions and actions can be initiated. The decision tree (Figure 5) with four decision attributes evaluates whether such a gap exists toward the target or current heading, replicating the avian “gap-first” decision process.

#### 3.2.1. Heading Direction Judgment

Analogous to pigeons requiring a destination, such as the nest, during flight, the position of the target point remains one of the factors to be consistently considered throughout the flight of a quadrotor. Therefore, it is necessary to judge based on the deviation between the current forward direction and the target point position. If the target point is not in the field-of-view cone of the current camera, it will be considered that the heading is deviated at this time.

(24) $$u_{t}^{1}=\{\begin{array}{cc} 1 & ,p_{c}^{\mathrm{target}}\in \left\|p_{c}^{\mathrm{target}}\right\|\cdot e^{i\cdot FOV_{c}}\\ 0 & ,p_{c}^{\mathrm{target}}\notin \left\|p_{c}^{\mathrm{target}}\right\|\cdot e^{i\cdot FOV_{c}} \end{array}$$

#### 3.2.2. Target-Point-Direction Obstacle Judgment

We select a high-confidence depth threshold $T_{d}$, satisfying $T_{d}\in d_{c}$, to segment the closed area occupied by the foreground obstacle $\Omega_{obs}$ and the flyable background area without obstacles $\Omega_{bg}$. The boundary contour of the obstacle is represented by $\partial \Omega_{obs}$.

(25) $$\{\begin{array}{l} \Omega_{obs}\cap \Omega_{bg}=\partial \Omega_{obs}\\ \Omega_{obs}\cup \Omega_{bg}=I_{t} \end{array}$$

Define $d\left(p_{uv}\right)$ as the minimum distance from a point to the obstacle boundary. The safety radius $R_{safe}$ is the maximum distance of the safety zone $\Omega_{safe}$. Then area $B_{R_{safe}}\left(p_{uv}\right)$ represents a closed neighborhood with $p_{uv}$ as the center and $R_{safe}$ as the radius.

(26) $$d\left(p_{uv}\right)=\min d\left(p_{uv},\partial \Omega_{obs}\right)$$

(27) $$R_{safe}=\max_{p_{uv}\in \Omega_{safe}}d\left(p_{uv}\right)$$

(28) $$B_{R_{safe}}\left(p_{uv}\right)=\left\{x\in \Omega_{img}|\left\|x-p_{uv}\right\|\leq R_{safe}\right\}$$

During flight, it is necessary to judge whether there are obstacles in the direction of the target point. If there is no obstacle interference between the own position and the target point, it means that obstacle avoidance is currently not required.

(29) $$u_{t}^{2}=\{\begin{array}{cc} 1 & \begin{array}{cc} ,\forall x\in B_{R_{safe}}\left(p_{uv}^{\mathrm{target}}\right) & ,I_{t}\left(x\right)<T_{d} \end{array}\\ 0 & \begin{array}{cc} ,\exists x\in B_{R_{safe}}\left(p_{uv}^{\mathrm{target}}\right) & ,I_{t}\left(x\right)\geq T_{d} \end{array} \end{array}$$

#### 3.2.3. Forward-Direction Obstacle Judgment

The obstacle judgment in the forward direction is similar to the obstacle judgment in the target-point direction. In addition to the target-point direction, the direction of the quadrotor is also a special direction. No matter what direction it is, it is at the center point in the pixel coordinate system. Currently, it is necessary to judge whether there is an obstacle in the direction of the center point. If there is no obstacle in front of the quadrotor, it means that there is no need to avoid obstacles without changing direction, which directly emulates the pigeon’s strategy of minimizing posture changes during flight to avoid unnecessary attitude adjustments, which conserves energy.

(30) $$u_{t}^{3}=\{\begin{array}{cc} 1 & \begin{array}{cc} ,\forall x\in B_{R_{safe}}\left(p_{uv}^{\mathrm{center}}\right) & ,I_{t}\left(x\right)<T_{d} \end{array}\\ 0 & \begin{array}{cc} ,\exists x\in B_{R_{safe}}\left(p_{uv}^{\mathrm{center}}\right) & ,I_{t}\left(x\right)\geq T_{d} \end{array} \end{array}$$

where $p_{uv}^{\mathrm{center}}={\left[\begin{array}{cc} COL/2 & ROW/2 \end{array}\right]}^{T}$ is the center position of the image.

#### 3.2.4. Gap Direction Judgment

During flight operations, the quadrotor may encounter not only common columnar obstacles but also wall-type obstacles. This can lead to a degradation in visual information quality, meaning the visual data obtained cannot match the richness available under ideal conditions. Under such circumstances, it becomes crucial to determine the direction of potential gaps and identify a navigable passage within the entire visual information framework that allows the quadrotor to pass through safely. In the absence of such a gap, the quadrotor should execute evasive maneuvers to exit the current field of view and explore alternative flight paths, which parallels the pigeon’s instincts when no viable gap is perceived.

(31) $$u_{t}^{4}=\{\begin{array}{cc} 1 & \begin{array}{ccc} \exists p_{uv}\in \Omega_{img} & ,\forall x\in B_{R_{safe}}\left(p_{uv}\right) & ,I_{t}\left(x\right)\geq T_{d} \end{array}\\ 0 & \begin{array}{ccc} \forall p_{uv}\in \Omega_{img} & ,\exists x\in B_{R_{safe}}\left(p_{uv}\right) & ,I_{t}\left(x\right)<T_{d} \end{array} \end{array}$$

In the actual computation process, the quadrotor’s safety region can be utilized for jump judgments. As mentioned above, pigeons are able to rapidly identify entire topological gaps as coherent regions through the lateral inhibition mechanism in the retina. Following this biological principle, our method focuses on identifying regions that meet threshold criteria, rather than individual pixels. Consequently, this approach significantly enhances operational speed and circumvents unnecessary repetitive searches. The extracted image information outcomes are systematically organized and documented as $S_{t}$ to filter unsafe actions through constraint judgment.

(32) $$S_{t}=\phi \left(I_{t}\left(\Omega_{img}\right)\right)={\left[\begin{array}{cccc} u_{t}^{1} & u_{t}^{2} & u_{t}^{3} & u_{t}^{4} \end{array}\right]}^{T}$$

### 3.3. Pigeon-Inspired Depth-Reasoning-Driven Action Decision

**Figure 6:**
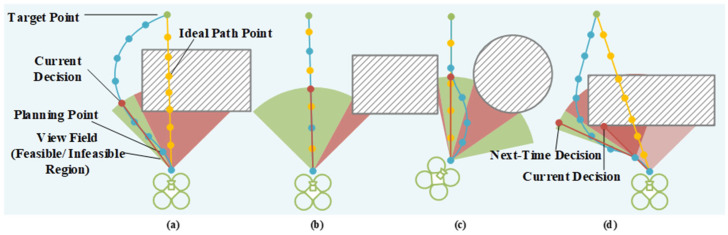
Schematic diagram of four quadrotor trajectory generation results. (**a**–**d**) The schematic results under different conditions.

Drawing inspiration from the pigeon’s ability to instantly select the safest and most efficient flight path among multiple visual gaps, our action decision module translates depth-derived safety fields into optimal trajectory directions. Based on the judgment criteria, we can distinguish between safe and unsafe regions. The tendency of pigeons to traverse gaps in a manner that minimizes changes to their posture can be modeled by a potential field that attains an extremum at the optimal location within the gap and gradually decays toward the boundaries. We further incorporate the target direction as an attractor, analogous to the bird’s homing instinct, resulting in a composite field that balances gap centering with goal progression. Within the safe region, it is necessary to devise a behavioral decision-making function that enables the quadrotor to select an optimal trajectory within the redundant space, as shown in Figure 6.

#### 3.3.1. Target-Point Direction Decision

To mathematically formalize this biological instinct, we map the visual saliency of the selected gap into a navigational potential field. Pigeons need to maintain their destination, such as their nest, during flight, which means that a centered gradient potential field function $J_{\mathrm{target}}$ must be created. This function will be used to enhance the spatial trajectory path of the quadrotor. This potential field function is based on a covariance matrix $R_{\mathrm{target}}$.

(33) $$\begin{array}{c} J_{\mathrm{target}}\left(p_{uv}\right)≜{\left(p_{uv}-p_{uv}^{\mathrm{target}}\right)}^{T}R_{\mathrm{target}}\left(p_{uv}-p_{uv}^{\mathrm{target}}\right)\\ ={\left\|p_{uv}-p_{uv}^{\mathrm{target}}\right\|}_{R_{\mathrm{target}}}^{2} \end{array}$$

(34) $$R_{\mathrm{target}}=\sigma_{\mathrm{target}}\sigma_{\mathrm{target}}^{T}=\left[\begin{array}{cc} \sigma_{\mathrm{target},1}^{2} & \sigma_{\mathrm{target},1}\sigma_{\mathrm{target},2}\\ \sigma_{\mathrm{target},2}\sigma_{\mathrm{target},1} & \sigma_{\mathrm{target},2}^{2} \end{array}\right]$$

#### 3.3.2. Center-Point Direction Decision

As mentioned earlier, pigeons have a path selection mechanism that minimizes attitude changes during flight, so a mathematical model for this part needs to be added to the optimization terms. Like the target point, define another potential field source. This part can be understood as combining the result with the current posture information of the quadrotor.

(35) $$\begin{array}{c} J_{center}\left(p_{uv}\right)≜{\left(p_{uv}-p_{uv}^{center}\right)}^{T}R_{center}\left(p_{uv}-p_{uv}^{center}\right)\\ ={\left\|p_{uv}-p_{uv}^{center}\right\|}_{R_{center}}^{2} \end{array}$$

(36) $$R_{\mathrm{center}}=\sigma_{\mathrm{center}}\sigma_{\mathrm{center}}^{T}=\left[\begin{array}{cc} \sigma_{\mathrm{center},1}^{2} & \sigma_{\mathrm{center},1}\sigma_{\mathrm{center},2}\\ \sigma_{\mathrm{center},2}\sigma_{\mathrm{center},1} & \sigma_{\mathrm{center},2}^{2} \end{array}\right]$$

We can also assign different weights σ to the two dimensions of the image, as shown in Figure 5c. This transforms the equipotential lines from circles into ellipses, thereby adjusting the emphasis on horizontal flight and climbing actions during flight.

#### 3.3.3. Gradient Safety Field

In addition to the quadcopter’s radius, this safety radius must also consider the distance from obstacles that is required due to airflow disturbances during flight. The obstacle avoidance behavior of pigeons is driven by the looming effect, which correlates with the rate of expansion of an object on the retina, enabling them to achieve smooth, oscillation-free flight trajectories even in confined spaces. Consequently, a gradual safety-field function $J_{safe}$ has been designed, where the potential field strength can be adjusted using a specific parameter $k_{safe}$.

(37) $$J_{safe}\left(p_{uv}\right)=\{\begin{array}{ll} f\left(\frac{d\left(p_{uv}\right)}{R_{safe}}\right)≜\exp\left(-k_{safe}\left(\frac{d\left(p_{uv}\right)}{R_{safe}}\right)\right) & ,p_{uv}\in \Omega_{safe}\\ 0 & ,otherwise \end{array}$$

The overall potential function is delineated as follows. Upon employing the proposed criteria to ascertain the necessity for obstacle avoidance, this potential function is utilized to derive the desired trajectory point, wherein the adjustable weight coefficients $\omega_{i}\left(i=1,2,3\right)$.

This potential function represents a vector that points from the target direction to the desired direction within the safe space, under the influence of the safety potential field emanating from the obstacle boundary. This approach ensures that the trajectory constraints are not merely geometric boundaries, but represent a dynamic ‘safety envelope’ that reacts to the visual flux, wherein mapping bridges the gap between discrete perception and continuous control.

(38) $$J_{potenial}=\omega_{1}\cdot J_{\mathrm{target}}+\omega_{2}\cdot J_{\mathrm{center}}+\omega_{3}\cdot J_{safe}$$

(39) $$p_{c}^{*}=K_{c}^{-1}p_{uv}^{*}=K_{c}^{-1}\cdot \underset{u,v}{\arg\min}\left(J_{total}\right)$$

The graph of this objective function is depicted in Figure 5d. While it is non-convex in its entirety, it exhibits local convexity. Provided that the initial value for the optimization is chosen as the target point position, the problem translates into a local convex optimization with an optimal solution.

### 3.4. Spatiotemporal Traversal Trajectory Generation

Once a pigeon selects a flight direction, it executes a smooth trajectory that respects its dynamic constraints. Our polynomial trajectory generation step corresponds to this motor planning phase, producing dynamically feasible paths that interpolate the waypoints derived from the potential field. The set of path points obtained in the above algorithm is $P=\left\{p_{0},p_{1},\ldots ,p_{M}\right\}$; the trajectory is divided into M segments, each of which is parameterized by the following L-order polynomial in $t\in \left[t_{k},t_{k+1}\right]$.

(40) $$J={\sum_{k=0}^{M-1}\int_{t_{k}}^{t_{k+1}}\left\|\frac{d^{3}p_{k}\left(t\right)}{dt^{3}}\right\|}^{2}dt$$

Equation (40) defines the polynomial representation of each trajectory segment $p_{k}\left(t\right)$, and order 3 is chosen to ensure the continuity of position, velocity, acceleration and jerk at segment boundaries. Minimizing jerk yields smooth, visually stable trajectories suitable for quadrotor flight. The objective function can be analytically expressed as a quadratic form with coefficients $a={\left[a_{0}^{0},\ldots ,a_{1}^{L},\ldots ,a_{M-1}^{L}\right]}^{T}$.

(41) $$L=a^{T}Q\left(L\right)a$$

where Q(L) is the block diagonal Hessian matrix associated with the polynomial order L. The trajectory satisfies the following constraints: path point constraints, derivative continuity constraints, and dynamic constraints.

(42) $$\{\begin{array}{l} p_{k}\left(t_{k}\right)=p_{k}^{start}\\ p_{k}\left(t_{k+1}\right)=p_{k}^{end}\\ \frac{d^{m}p_{k-1}\left(t_{k}\right)}{dt^{m}}=\frac{d^{m}p_{k}\left(t_{k}\right)}{dt^{m}},\;m=0,1,2\\ \left\|v\left(t\right)\right\|=\left\|\frac{dp\left(t\right)}{dt}\right\|\leq v_{\max}\\ \left\|a\left(t\right)\right\|=\left\|\frac{d^{2}p\left(t\right)}{dt^{2}}\right\|\leq a_{\max} \end{array}$$

Equation (42) summarizes the constraints that the trajectory must satisfy: waypoint constraints, continuity constraints and dynamic feasibility constraints. Together, these three equations define a standard minimum-snap trajectory optimization problem, which we solve using quadratic programming and time adjustment following established methods. In addition, the obstacle avoidance constraint is implicit in the path point set P. As shown in Figure 4b, during the planning process, the limited space is divided into several safe spaces.

(43) $$\begin{array}{cc} P_{safe}=B_{ℛ}\left(P\right) & R= \end{array}v\left(t\right)\cdot \Delta t_{c}\pm R_{safe}$$

where $\Delta t_{c}$ is the sampling interval of the camera. In the final trajectory, we use the decoupled optimization of time and space

(44) $$\underset{a,T}{\min J}\left(a,L\right)+\omega_{J}\sum_{k=0}^{M-1}\Delta t_{k}$$

where $\omega_{J}$ is the optimization weight coefficient.

## 4. Experimental Results and Analysis

To validate the proposed framework, comprehensive experiments are conducted in both simulation environments and real-world scenarios. All tests emphasized autonomous navigation in unmapped 3D spaces without prior information.

### 4.1. System Experiment

Simulations are executed in Gazebo, which models complex environments. The quadcopter equipped with a depth camera performs obstacle avoidance flight in a space full of obstacles and design a Visual–Inertial Odometer for real-time positioning during the flight. The algorithm and physical structure parameters are set as listed in Table 1, which includes the basic structural parameters of the camera and the basic physical parameters of the UAV, all of which are set to appropriate values. The parameter weights of the method proposed in this paper are fitness values obtained through experimental tuning to ensure the best trade-off between smoothness and collision avoidance. The safety margin pixel value is calibrated based on the depth sensor resolution and is positively correlated with the safety radius of the gap expansion required to identify the gap at flight speed. To rigorously evaluate the proposed pigeon-inspired depth-reasoning-driven decision framework, we conducted comparative experiments against FASTER [31] and the Artificial Potential Field (APF) method. It is important to note that for the purposes of these comparative baselines, both FASTER and APF were provided with pre-processed local maps or explicit obstacle position information as inputs to ensure their standard operational requirements. In contrast, our proposed method operates directly on raw, unprocessed depth information without any prior knowledge or map construction.

**Figure 7:**
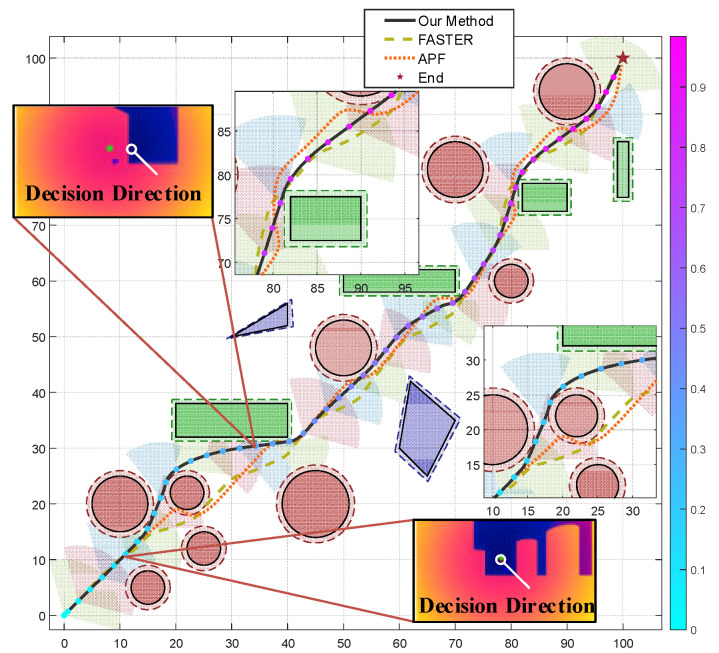
The 2D flight trajectory image of the quadrotor. The fan-shaped area is part of the visual range detected by the camera.

**Figure 8:**
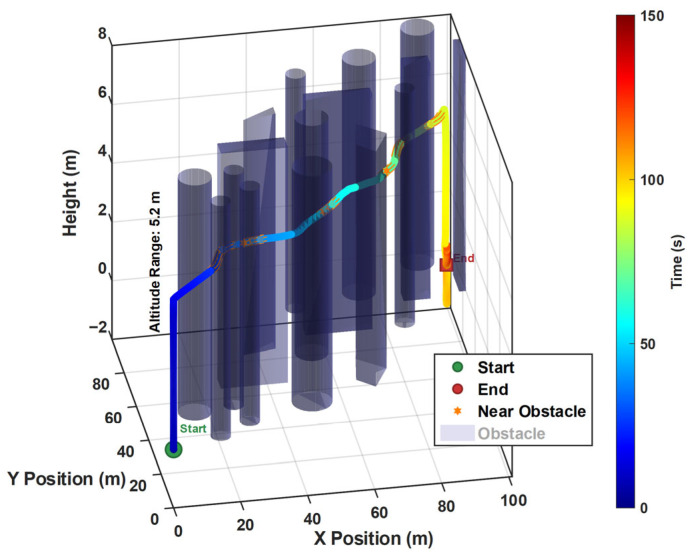
The 3D image of the quadcopter flying through space with obstacle avoidance. The translucent cylinders are obstacles, and the colored curves are the flight paths of the quadrotors over time.

Figure 7 and Figure 8 present the flight path of the quadrotor in 2D and 3D, respectively, combining real-time visual information to perform dynamic obstacle avoidance, and mark the location of obstacles in the environment. The color image of the camera is used for identification and positioning, and the depth map is used for obstacle avoidance and positioning. Near the coordinate (10, 10, 5), the depth information detects the existence of an obstacle, as shown in the depth map in Figure 7. This causes a significant deviation in the original path, forming a local avoidance trajectory. Similar avoidance is also reflected in the flat obstacle near the point (35, 30, 5). And the avoidance process is always kept outside the safe distance.

Despite the obstacle avoidance maneuver, the overall path is relatively smooth, without violent reentry or oscillation, indicating that the path planning algorithm takes into account flight efficiency while ensuring safety. This figure strongly proves that the fused visual information, especially the accurate distance perception provided by the depth map, is the key to achieving robust, real-time obstacle avoidance and traversal flight.

The average values from multiple runs of the experiment are shown in Table 2, where smoothness $=1/\left(1+\bar{\kappa }\right)\in \left(0,1\right]$; $\bar{\kappa }$ is the average curvature along the trajectory weighted by arc length and higher values indicate smoother paths. While FASTER and APF achieve smooth obstacle avoidance using the provided local maps, they incur significant hidden costs in real-world scenarios due to the necessity of map building (e.g., voxel grid or ESDF updates). Importantly, the reported calculation time corresponds exclusively to its trajectory optimization stage; map construction was performed offline prior to planning and is not included at this time. This difference arises from the fundamental algorithmic distinction: FASTER performs iterative gradient-based optimization on a high-resolution ESDF grid, while our method directly reasons on raw depth pixels using a lightweight potential field, requiring no discretization or iterative solver. In contrast, APF achieves the lowest computation time but suffers from oscillations and local minima due to its reactive nature. Our method eliminates this dependency, directly translating pixel-level depth reasoning into control constraints, which is inherently more robust to the latencies typically introduced by mapping threads in unmapped 3D environments. The experimental results in Figure 7 illustrate that, while FASTER produces more optimal paths by exploiting known obstacle geometries, its performance is strictly bound by the accuracy of the map. In contrast, our approach demonstrates comparable safety despite the lack of a local map. By reasoning directly from the raw depth field, our method avoids the mapping lag and potential discretization errors inherent in voxel-based representations. While APF remains a fast reactive baseline, its reliance on a clear potential field gradient often leads to suboptimal oscillations, whereas our depth-reasoning logic generates smoother, more dynamically feasible trajectories by considering the 3D geometric constraints of the FOV. Please refer to Supplementary Material Figure S1 for the overall flowchart of the method.

### 4.2. Real-World Experiment

**Figure 9:**
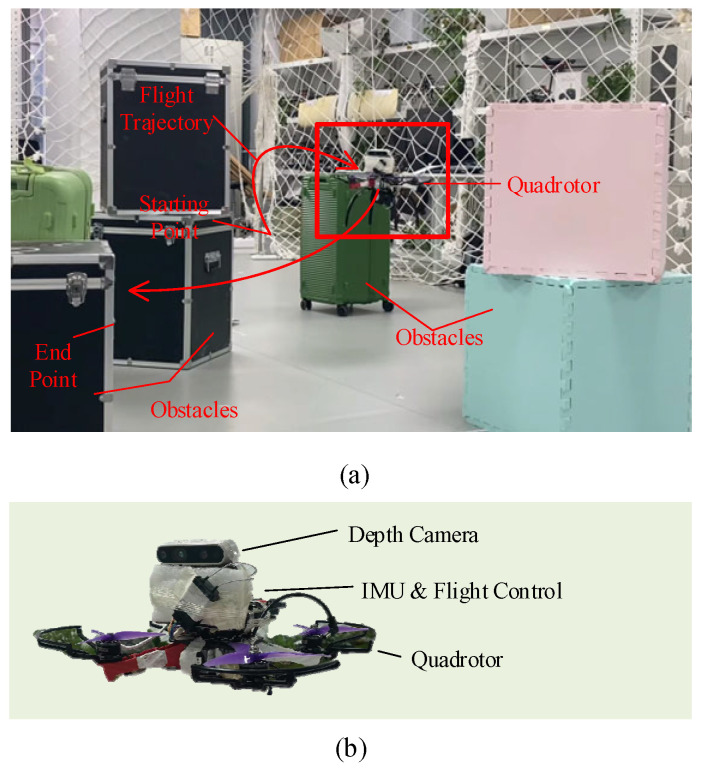
Flight verification test of actual quadrotor. (**a**) Test site and process. (**b**) Quadrotor with depth camera we used.

In addition to the simulation experiment, we conducted actual flight verification in our existing environment under multiple conditions. The experimental site and equipment are shown in Figure 9. In our real-world flight experiments, the quadrotor platform is equipped with an Intel Jetson Xavier NX as the primary onboard computer for high-level tasks, including VIO processing and the proposed pigeon-inspired depth-reasoning-driven decision algorithm. A CUAV X7+ flight controller is utilized for low-level attitude and position control. To ensure the reliability of depth information—upon which our map-less trajectory planning heavily relies—we selected a relatively moderate and structured experimental environment. This choice was made to maintain the stability of the raw depth data provided by the stereo camera, thereby minimizing the impact of severe sensor noise and allowing for a more focused evaluation of the planning algorithm’s decision-making logic and real-time performance in unmapped 3D spaces. The depth camera we used was Intel’s D435i camera, and its parameter information is shown in Table 3. The depth camera receives information about the surrounding environment and generates decision information to guide the quadrotor to fly quickly through a space full of obstacles.

**Figure 10:**
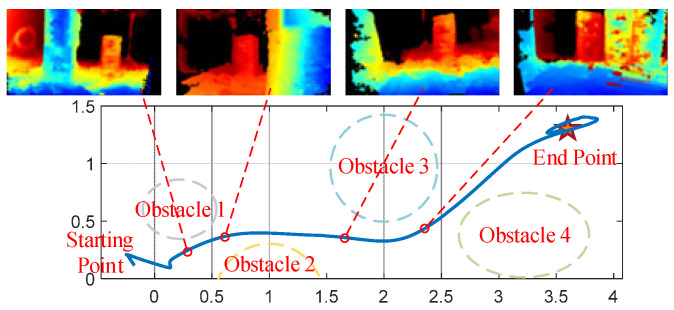
Actual obstacle avoidance flight trajectory and depth information image of quadrotor aircraft. In the depth map, the color spectrum transitions continuously from blue (nearest) to red (farthest).

**Figure 11:**
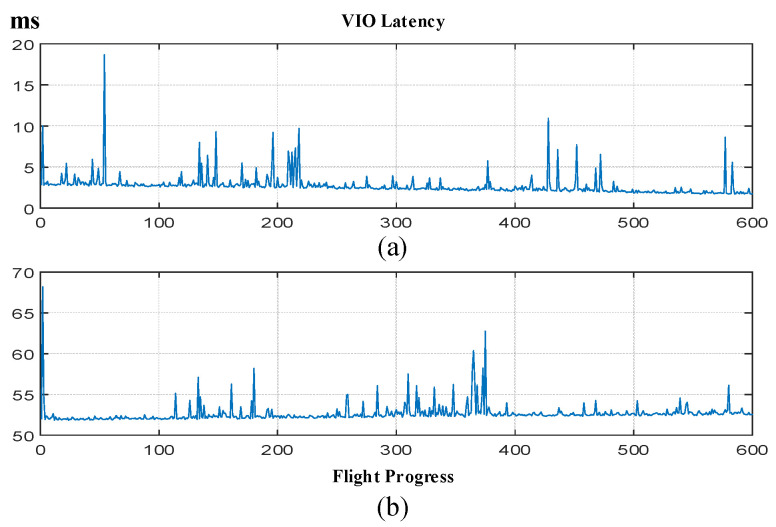
Delay of VIO during flight and mission process. (**a**) ORB-based VIO. (**b**) SuperGlue-based VIO.

As shown in Figure 10, the quadrotor successfully navigated through a cluttered environment containing four obstacles with an average spacing of 1 m. Despite the constrained space, the trajectory remained smooth with a total path length of 4.4 m. As shown in Figure 11, the VIO delay characteristics reveal robust performance under real-flight conditions, with an average processing latency of 52.6 ms. And during the entire verification process, the counter is not reset, which proves its robustness.

**Figure 12:**
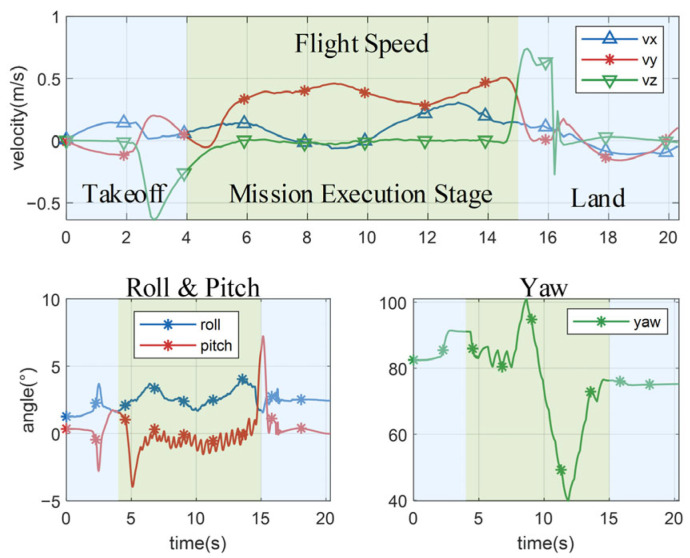
The attitude curve and speed curve of the quadrotor during obstacle avoidance and crossing flight.

Similar to the results in the simulation, the flight speed and attitude angle of the quadrotor can be maintained within a stable range during the actual flight to ensure a smooth path as shown in Figure 12. During the actual flight, due to the limitation of the site size, in order to ensure the safety of the flight, we limited the speed of the quadrotor. The results showed that the quadrotor can still maintain a flight speed of about 0.5 m/s while performing the obstacle avoidance and crossing flight mission. After multiple actual flight verifications, the results show that the proposed pigeon-inspired depth-reasoning-driven decision framework is designed for high-speed traversal in unmapped environments where computational efficiency is paramount. However, its performance is subject to certain constraints and environmental factors.

As demonstrated in practical validation, our proposed pigeon-inspired depth-reasoning-driven decision fundamentally relies on the quality of raw depth inputs. The entire decision-making pipeline, from gap detection to safety field construction, operates directly on depth measurements without explicit map filtering or temporal accumulation. Consequently, the performance of the framework is inherently sensitive to the accuracy of the depth sensor. In environments with highly reflective surfaces or extreme lighting conditions that cause depth holes or significant noise, the depth-reasoning module may generate suboptimal or overly conservative constraints. Although the filtering and weighting-based coupling methods employed in this approach can mitigate such dependency to a certain extent, it must be acknowledged that the reliability of the depth data acquired by the sensor remains a critical factor. Although we employ SuperGlue to enhance matching robustness, any significant drift in state estimation will directly translate into errors in the world-frame projection of depth constraints, potentially leading to collision risks during high-speed maneuvers.

## 5. Conclusions

This paper presents a pigeon-inspired depth-reasoning-driven decision framework designed to overcome the computational bottlenecks and mapping latencies inherent in autonomous quadrotor flight in unmapped 3D spaces. The framework directly emulates three key avian navigation strategies: gap selection via instantaneous depth gradients, path selection that minimizes posture changes, and a safety field driven by the looming effect. By bypassing traditional spatial discretization and explicit map construction, the proposed system establishes a direct link between raw sensory depth perception and trajectory generation.

First, to realize the gap selection mechanism, the framework segments raw depth images in real time to identify passable topological gaps without constructing a global map. Second, to replicate the path selection strategy that minimizes posture changes, we construct a gradient potential field that guides the quadrotor toward the optimal position of the selected gap. This encourages smooth, minimally oscillatory trajectories, analogous to the pigeon’s tendency to maintain flight stability during gap traversal. Third, to implement the looming-effect-driven safety field, we define a non-linear repulsive potential whose strength increases inversely with distance to obstacles.

Together, these three biologically inspired components form a constraint-fusion planning strategy that combines hard safety limits with soft optimization to generate dynamically feasible trajectories from sparse waypoints, replicating the instantaneous trade-off between collision avoidance and flight efficiency observed in nature. To enable this framework in practice, we integrate a robust perception backbone that combines depth-guided visual–inertial odometry with deep-learning-based feature matching, providing the necessary state estimation stability under aggressive maneuvers.

Experimental validation confirms the framework’s effectiveness in unmapped 3D spaces. Future efforts will focus on exploring more profound biological mechanisms and enhancing the system’s capability to handle highly dynamic obstacles, further optimizing computational efficiency for onboard deployment, and extending its application to large-scale, long-duration autonomous exploration missions.

## Figures and Tables

**Table 1:** The algorithm and physical structure parameters.

Parameters	Values	Parameters	Values
FOV	120°	$T_{d}$	100
$d_{\min}$	0 m	$d_{\max}$	10 m
$\omega_{1}$	0.0008	$\omega_{2}$	0.001
$\omega_{3}$	100	$R_{safe}$	30 px
$\Delta t_{c}$	0.2 s	$\Delta t_{k}$	0.2 s
$\omega_{J}$	1.5	$\omega_{\max}$	45°/s
$a_{\min}$	−0.5 m/s^2^	$a_{\max}$	0.2 m/s^2^
$v_{\min}$	0 m/s	$v_{\max}$	1 m/s

**Table 2:** Algorithm comparison results in simulation.

Algorithm	Trajectory Length	Track Time	Smoothness	Calculation Time	Minimum Distance	Proportion Less than 0.85 m
Our Method	147.0 m	149.4 s	0.961	30.836 s	0.806 m	0.40%
FASTER	145.8 m	148.2 s	0.943	143.798 s	1.038 m	0.00%
APF	151.4 m	153.8 s	0.919	0.087 s	0.309 m	2.73%

**Table 3:** Depth camera parameter information.

Parameter	Value
Depth Detection Range	0.2~10 m
Binocular Baseline	50 mm
Depth Stream Frequency	Up to 90 FPS

## Data Availability

Data Availability Statement: The original contributions presented in the study are included in the article/Supplementary Material.

## Supplementary Materials

The following supporting information can be downloaded at: https://www.mdpi.com/article/10.3390/biomimetics11040283/s1, Figure S1: Pigeon-Inspired Depth-Reasoning-Driven Decision Framework for Quadrotors Diagram.
